# Effects of Flavonoids from French Marigold (Florets of *Tagetes patula* L.) on Acute Inflammation Model

**DOI:** 10.1155/2013/309493

**Published:** 2013-09-22

**Authors:** Ken Yasukawa, Yoshimasa Kasahara

**Affiliations:** ^1^School of Pharmacy, Nihon University, Chiba 274-8555, Japan; ^2^The Yamagata Prefectural Institute of Public Health, Yamagata 990-0031, Japan

## Abstract

The major components patuletin and patulitrin were isolated from French marigold (florets of *Tagetes patula*). Patuletin and patulitrin were found to inhibit acute inflammation in mice. Oral administration of patuletin and patulitrin significantly suppressed hind-paw edema induced by carrageenin and histamine, while topical application of patuletin and patulitrin significantly inhibited ear edema induced by 12-*O*-tetradecanoylphorbol-13-acetate and arachidonic acid. Thus, oral and topical administration of patuletin and patulitrin inhibited acute inflammation in mice. These results suggest the anti-inflammatory efficacy of French marigold.

## 1. Introduction

French marigold (florets of *Tagetes patula*) has been used internally for indigestion and externally for sore eyes and rheumatism. The plant, a member of the family Asteraceae, is a common cultivated ornamental. In general, these flowers are used as a coloring or flavoring agent in food and have potential medical use against inflammatory diseases of the skin, as well as insecticidal activity [1, 2]. Previous phytochemical investigations on French marigold have resulted in the isolation of carotenoids [3, 4], flavonoids [5, 6], and monoterpenoids [7]. In our previous studies, we reported the anti-inflammatory action of French marigold [8, 9], and in this paper, we report the inhibitory effects of the flavonol patuletin and its glucoside from methanol extracts of French marigold on acute inflammation models, such as the hind-paw edema induced by **γ**-carrageenin and histamine and ear edema induced by arachidonic acid (AA) and 12-*O*-tetradecanoylphorbol-13-acetate (TPA).

## 2. Materials and Methods

### 2.1. Materials

*Tagetes patula* L. was cultivated at the Yamagata Prefectural Horticultural Experiment Station in Yamagata, Japan. A voucher specimen (ER-070) of *Tagetes patula* L. (Asteraceae) was deposited in the Herbarium of The Yamagata Prefectural Institute of Public Health, Yamagata-shi, Yamagata, Japan.

### 2.2. Extraction and Separation

Raw flower petals of *T. patula* L. (5 kg) were subjected to extraction twice with MeOH (5 L) at room temperature. Solvents were evaporated *in vacuo* to dryness, and an extract was obtained (yield: 3.9%). MeOH extract (35 g) was partitioned between EtOAc−H_2_O (1 : 1). The EtOAc extract (7.2 g) was partitioned between *n*-hexane−MeOH−H_2_O (19 : 19 : 2), which afforded an *n*-hexane fraction (0.94 g) and a MeOH−H_2_O fraction (6.26 g). On the other hand, the H_2_O solution was partitioned between *n*-BuOH−H_2_O (1 : 1), yielding an *n*-BuOH extract (4.74 g) and an H_2_O extract (22.04 g), respectively. The MeOH−H_2_O fraction (6 g) was subjected to CC on Sephadex LH-20 (100 g) using MeOH. The potent active fraction was subjected to same CC using 90% MeOH, which yielded two active fractions, fr-3 and fr-7 (yields: 2.5 g and 1.6 g). Fr-3 was recrystallized to give compound-1 (1.8 g) from 90% ethanol, and fr-7 was recrystallized to give compound-2 (0.9 g) from ethanol. Compound-1 and Compound -2 were identified as patuletin and patulitrin by NMR and mass spectrometry [10, 11].

### 2.3. Chemicals

The following chemicals were purchased: **γ**-carrageenin from Zushikagaku Laboratory, Inc. (Kanagawa, Japan); phenylbutazone, cyproheptadine, and arachidonic acid from Sigma Chemical Co, (St. Louis, MO); TPA from Chemicals for Cancer Research, Inc. (Chicago, IL); and histamine, methanol, and acetone from Wako Pure Chemical Industries, Ltd. (Osaka, Japan).

### 2.4. Animals

Experiments were performed in accordance with the Guidelines of the Institutional Animal Care and Use Committee of the School of Pharmacy, Nihon University (Chiba, Japan). Female ICR mice (age, 7 weeks) and male ddY mice (22~26 g) were purchased from Japan SLC Inc. (Shizuoka, Japan) and were housed in an air-conditioned specific pathogen-free room (22-23°C) illuminated from 08:00−20:00. Food and water were available *ad libitum*.

### 2.5. *γ*-Carrageenin-Induced Mouse-Paw Edema

The inhibitory activity of flavonoids on carrageenin-induced mouse-paw edema was assessed according to the method of Tsurufuji et al. [12]. Briefly, mice were treated orally with flavonoids (each 5, 15 and 50 mg/kg) or vehicle (10 mL/kg). Samples were administered orally 30 min before injection of 25 *μ*L of a 2% suspension of carrageenin in physiological saline solution into the subcutaneous tissues of the right hind paw. The left hind paw was injected in the same manner with 25 *μ*L of physiological saline solution. Edema measurements were made using a dial thickness gauge (Ozaki MFG Co., Ltd., Tokyo, Japan) at intervals of 1, 2, 3, 4, 5, and 6 hours after carrageenin injection.

### 2.6. Hind-Paw Edema Induced by Histamine in Mice

The assay method was based on the report by Tsurufuji et al. [12]. Mice in groups of five at each dose level received flavonoids (each 5, 15, and 50 mg/kg) in water (10 mL/kg, *p.o*.). At 30 min after flavonoid administration, mice were injected with histamine (5 *μ*g) in physiological saline solution (5 *μ*L) into the subplantar tissue of the right hind paw, and with saline solution (5 *μ*L) into that of the left hind paw. The difference in foot pad thickness between the right and left paws was then measured with a dial thickness gauge every 6 min.

### 2.7. Assay of TPA- and AA-Induced Inflammation in Mice

Assays were conducted in accordance with the methods reported by Yasukawa et al. [13]. TPA (1 *μ*g) or AA (1 mg) dissolved in acetone (20 *μ*L) was applied to the right ear only of mice by means of a micropipette. A volume of 10 *μ*L was delivered to both the inner and outer surfaces of the ear. Flavonoids (each 0.04, 0.2, and 1.0 mg/ear) or vehicle, MeOH-CHCl_3_ (1 : 1, 20 *μ*L) or MeOH–CHCl_3_–H_2_O (2 : 1 : 1, 20 *μ*L) were topically applied 30 min before TPA or AA treatment. Ear thickness was determined with a pocket thickness gauge (Mitsutoyo Co., Ltd., Tokyo, Japan) having a range of 0–9 mm, graduated at 0.01-mm intervals and modified such that the contact surface area was increased to reduce loading when applied to the tip of the ear. Ear thickness was measured before treatment (*a*) and at 6 (1) h after TPA (AA) treatment (*b* = TPA (AA) plus vehicle; *b*′ = TPA (AA) plus sample). The following values were then calculated:

(i) edema A induced by TPA (AA) plus vehicle (*b* − *a*);
(ii) edema B induced by TPA (AA) plus sample (*b*′ − *a*),
(iii) inhibitory  rate  (%) = [(edema  A − edema  B)/edema  A] × 100,
(iv) each value was the mean of individual determinations from four mice.

### 2.8. Data Analysis

Data are expressed as means ± standard deviation and were analyzed using Prism statistical software. Differences between groups (Figures 2, 3, 4, 5, 6, and 7) were analyzed by one-way ANOVA followed by correction with Tukey-Kramer test.

## 3. Results

### 3.1. Effects of Flavonoids from *Tagetes patula* on *γ*-Carrageenin-Induced Hind-Paw Edema in Mice

**Figure 1:**
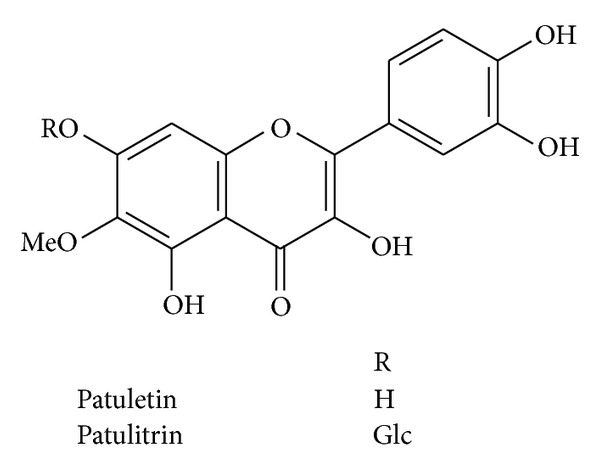
The structures of flavonoids from *Tagetes putula*.

**Figure 2:**
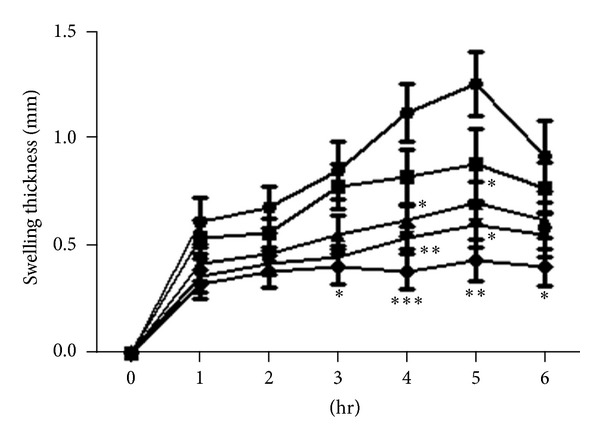
Inhibitory effect of patuletin on carrageenin-induced hind-paw edema in mice. Drugs were administered *p*.*o*. 30 min before carrageenin (2%, 25 *μ*L). *⚫*: control; ■: patuletin 5 mg/kg; ▲: patuletin 15 mg/kg; *▼*: patuletin 50 mg/kg; ♦: phenylbutazone 100 mg/kg. Significantly different from the control, *P < 0.05, **P < 0.01, or ***P < 0.001. Each value represents the mean ± SD of 5 mice.

**Figure 3:**
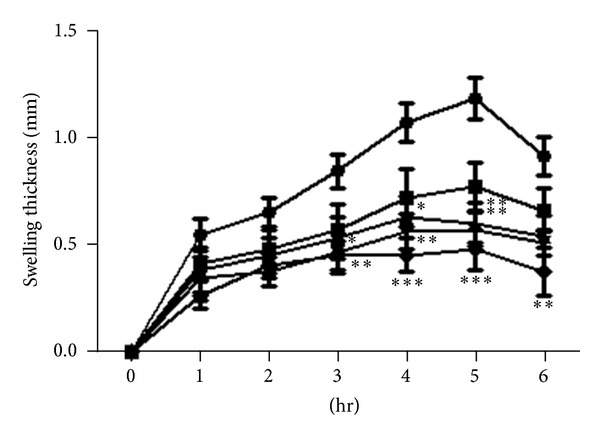
Inhibitory effect of patulitrin on carrageenin-induced hind-paw edema in mice. Drugs were administered *p*.*o*. 30 min before carrageenin (2%, 25 *μ*L). *⚫*: control; ■: patulitrin 5 mg/kg; ▲: patulitrin 15 mg/kg; *▼*: patulitrin 50 mg/kg; ♦: phenylbutazone 100 mg/kg. Significantly different from the control, *P < 0.05, **P < 0.01, or ***P < 0.001. Each value represents the mean ± SD of 5 mice.

Oral administration of patuletin and patulitrin suppressed carrageenin-induced edema in a dose-dependent manner. At 15 mg/kg and 50 mg/kg patuletin, edematization was significantly suppressed and the inhibition rates were 41–52% at 4-5 h. At 50 mg/kg patulitrin, hind-paw edema was suppressed at 3–5 h after administration, and inhibition rates were 45–52%. At 15 mg/kg patulitrin, hind-paw edema was suppressed at 4-5 h, while 10 mg/kg phenylbutazone suppressed edematization by 47–60% at 3–6 h (Figures 2 and 3). Patuletin and patulitrin suppressed carrageenin-induced edema in a dose-dependent manner. However, these compounds were less effective than phenylbutazone (100 mg/kg) (Figure 1).

### 3.2. Effects of Flavonoids from *Tagetes patula* on Histamine-Induced Hind-Paw Edema in Mice

**Figure 4:**
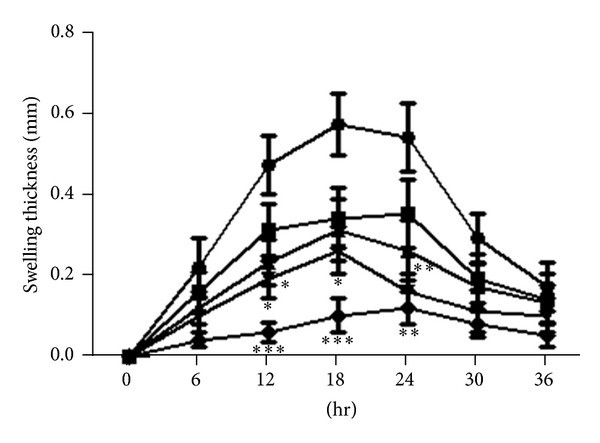
Inhibitory effect of patuletin on histamine-induced hind-paw edema in mice. Drugs were administered *p*.*o*. 30 min before histamine (5 *μ*g). *⚫*: control; ■: patuletin 5 mg/kg; ▲: patuletin 15 mg/kg; *▼*: patuletin 50 mg/kg; ♦: cyproheptadine 10 mg/kg. Significantly different from the control, *P < 0.05, **P < 0.01, or ***P < 0.001. Each value represents the mean ± SD of 5 mice.

**Figure 5:**
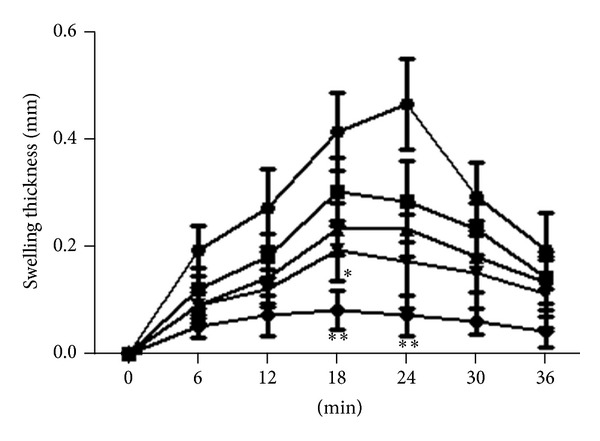
Inhibitory effect of patulitrin on histamine-induced hind-paw edema in mice. Drugs were administered *p*.*o*. 30 min before histamine (5 *μ*g). *⚫*: control; ■: patulitrin 5 mg/kg; ▲: patulitrin 15 mg/kg; *▼*: patulitrin 50 mg/kg; ♦: cyproheptadine 10 mg/kg. Significantly different from the control, *P < 0.05 or **P < 0.01. Each value represents the mean ± SD of 5. Significantly different from the control, *P < 0.05, **P < 0.01, or ***P < 0.001. Each value represents the mean ± SD of 5 mice.

Figures 4 and 5 illustrate the time course of histamine-induced hind-paw edema in mice. At 50 mg/kg patuletin, edematization was significantly suppressed and inhibition rates were 54–70% at 12–24 min. At 50 mg/kg patulitrin, hind-paw edema was suppressed at 18 min after administration. Patuletin suppressed histamine-induced edema in a dose-dependent manner. However, these compounds were less effective than cyproheptadine (10 mg/kg) (Figure 1).

### 3.3. Effects of Flavonoids from *Tagetes patula* on TPA-Induced Ear Edema in Mice

**Figure 6:**
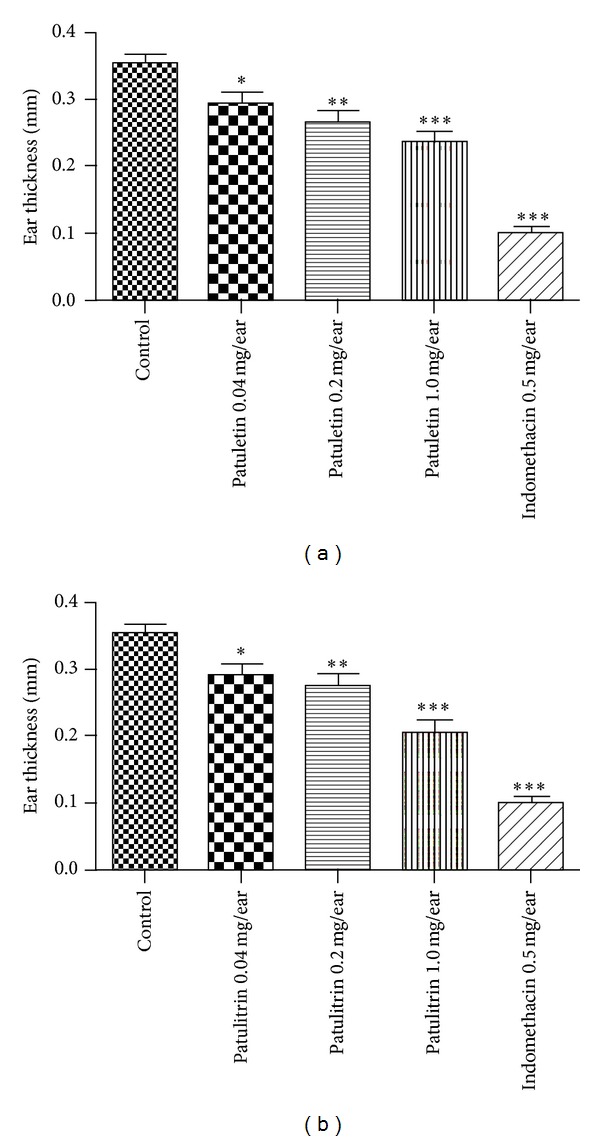
Inhibitory effect of patuletin and patulitrin on TPA-induced ear edema in mice. Drugs were administered topically 30 min before TPA (1 *μ*g). Significantly different from the control, *P < 0.05, **P < 0.01, or ***P < 0.001. Each value represents the mean ± SD of 4 mice.

Figure 6 illustrates the inhibitory effects on TPA-induced ear edema in mice. Patuletin and patulitrin suppressed TPA-induced ear edema in a dose-dependent manner. In comparison with a standard drug, these compounds were less effective than indomethacin (0.5 mg/ear).

### 3.4. Effects of Flavonoids from *Tagetes patula* on AA-Induced Ear Edema in Mice

**Figure 7:**
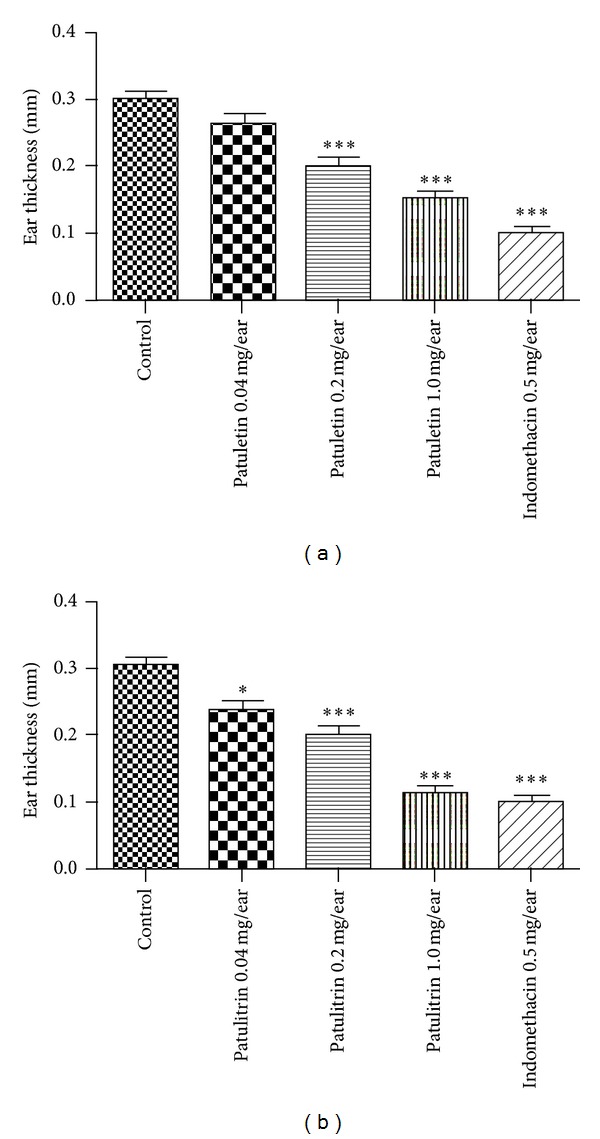
Inhibitory effect of patuletin and patulitrin on AA-induced ear edema in mice. Drugs were administered topically 30 min before AA (1 mg). Significantly different from the control, *P < 0.05, **P < 0.01, or ***P < 0.001. Each value represents the mean ± SD of 4 mice.

Figure 7 illustrates the inhibitory effects of AA-induced ear edema in mice. Patuletin and patulitrin inhibited AA-induced edema in a dose-dependent manner. However, these compounds were less effective than indomethacin (0.5 mg/ear).

## 4. Discussion

The flavonoids patuletin and patulitrin were isolated as major components from French marigold. Carrageenin-induced hind-paw edema is used as an animal model of acute inflammation and is thought to represent the early phase of inflammation. Oral administration of patuletin and patulitrin inhibited edematization in a dose-dependent manner, although 50 mg/kg patuletin and patulitrin suppressed hind-paw edema less effectively than 100 mg/kg phenylbutazone, an anti-inflammatory drug. Furthermore, the effects of patuletin and patulitrin on edema induced by histamine were investigated. Patuletin and patulitrin significantly inhibited histamine-induced hind-paw edema in a dose-dependent manner. The above findings clarified that patuletin and patulitrin are effective in treating acute inflammation by suppressing the chemical mediators of inflammation. These results suggest that the active components in the anti-inflammatory effects of French marigold are patuletin and patulitrin.

French marigold has been applied to the human foot to decrease inflammation caused by corns, calluses, hallux valgus, and ulcers and to reduce the associated pain [3]. Therefore, we examined the anti-inflammatory effects of patuletin and patulitrin after topical administration. Patuletin and patulitrin inhibited TPA- and AA-induced inflammatory ear edema in mice. These results suggest that the active components in the anti-inflammatory effects after the topical administration of French marigold are patuletin and patulitrin.

Some flavonoids have been found to inhibit inflammation in several experimental animal models [14]. They also inhibit the expression of inducible nitric oxide synthase (iNOS), cyclooxygenase (COX), and lipoxygenase (LOX), with subsequent decreases in nitric oxide (NO), prostanoids, and leukotrienes, as well as other mediators of the inflammatory process such as cytokines, chemokines, or adhesion molecules [15]. Body cells and tissues are continuously threatened by the damage caused by free radicals and reactive oxygen species, which are produced during normal oxygen metabolism or are induced by exogenous factors [16]. On evaluation of the 1,1-diphenyl-2-picrylhydrazyl (PDDF), radical-scavenging activity of patuletin and patulitrin exhibited potent radical-scavenging activities with IC_50_ values of 4.3 and 10.17 *μ*g/mL, respectively [17]. In addition, free radicals can attract various inflammatory mediators, contributing to a generalized inflammatory response and tissue damage. It has been reported that patuletin inhibits LOX [18]. Indeed, patuletin and patulitrin are powerful *in vitro* antioxidants, being able to scavenge a wide range of free radical species, as well as to inhibit their formation [19].

This is the first report to find that patuletin and patulitrin inhibit acute inflammation induced by carrageenin, histamine, TPA, and AA in mice.
